# PPAR*α* Enhances Cancer Cell Chemotherapy Sensitivity by Autophagy Induction

**DOI:** 10.1155/2018/6458537

**Published:** 2018-11-04

**Authors:** Mengli You, Jiaming Gao, Jianhua Jin, Yongzhong Hou

**Affiliations:** ^1^Department of Oncology, The Affiliated Wujin People's hospital, Jiangsu University, Changzhou, Jiangsu Province, China; ^2^Institute of Life Science, Jiangsu University, Zhenjiang, Jiangsu Province, China

## Abstract

PPAR*α* (peroxisome-proliferator-activated receptor *α*) plays a critical role in regulation of inflammation and cancer, while the regulatory mechanism of PPAR*α* on cancer cell autophagy is still unclear. Here we found that PPAR*α* enhanced autophagy in HEK293T, SW480, and Hela cell lines, which was independent of PPAR*α* transcription activity. PPAR*α* induced antiapoptotic Bcl2 protein degradation resulting in release of the Beclin-1/VPS34 complex. Consistently, silenced PPAR*α* reversed this event. PPAR*α*-induced autophagy significantly inhibited tumor growth and enhanced SW480 cancer cell sensitivity to chemotherapy drugs. Moreover, PPAR*α* agonist increased SW480 cancer cell chemotherapy sensitivity. These findings revealed a novel mechanism of PPAR*α*/Bcl2/autophagy pathway suppressed tumor progression and enhanced chemotherapy sensitivity, which is a potential drug target for cancer treatment.

## 1. Introduction

As one of the nuclear hormone receptor family, peroxisome-proliferator-activated receptor *α* (PPAR*α*) is a ligand-activated transcription factor. Ligand binding and activated PPAR*α* heterodimerizes with RXRs (Retinoid X receptors) lead to binding peroxisome-proliferator response element (PPRE: AGGTCA N AGGTCA, N is any nucleic acid) that regulates the target gene expression, which is involved in atherosclerosis, diabetes, obesity, inflammation, and cancer [1–7]. Clinical observation shows that expression of PPAR*α* contributes the survival of breast and ovarian cancer [8, 9]. The synthetic ligands of PPAR*α* including fenofibrate, clofibrate, and wyeth14,643 suppress cancer cell proliferation [2, 5]. As a nuclear receptor, PPAR*γ* induces NF*κ*B/p65 and MUC1-C ubiquitination and degradation independent of its transcription activity [2, 3]. Similarly, PPAR*α* serves as E3 ligase to induce Bcl2 ubiquitination and degradation leading to increased cancer cell apoptosis in response to chemotherapeutic agents [6]. As antiapoptotic protein, Bcl2 inhibits autophagy signaling by binding to Beclin-1 to inhibit Beclin-1/VPS34 complex [10]. Autophagy delivers cytoplasmic materials (proteins, lipids, etc.) or organelles (mitochondria, nucleus, etc.) into lysosomes for degradation, which is also a progress of nutrient recycling [11]. Autophagy contributes cancer cell survival during nutrient deprivation; however, cancer cells consume all of the cellar components resulting in cell death [11, 12]. Other reports show that ligand-activated PPAR*α* increases autophagy of AML12 cells or livers via PPAR*α*-mediated autophagy-associated gene expressions [13], while here we found that PPAR*α* induced cancer cell autophagy independent of its transcription activity by release of Beclin-1/VPS34 complex.

## 2. Results

### 2.1. PPAR*α* Induces Autophagy Independent of Its Transcription Activity

**Figure 1:**
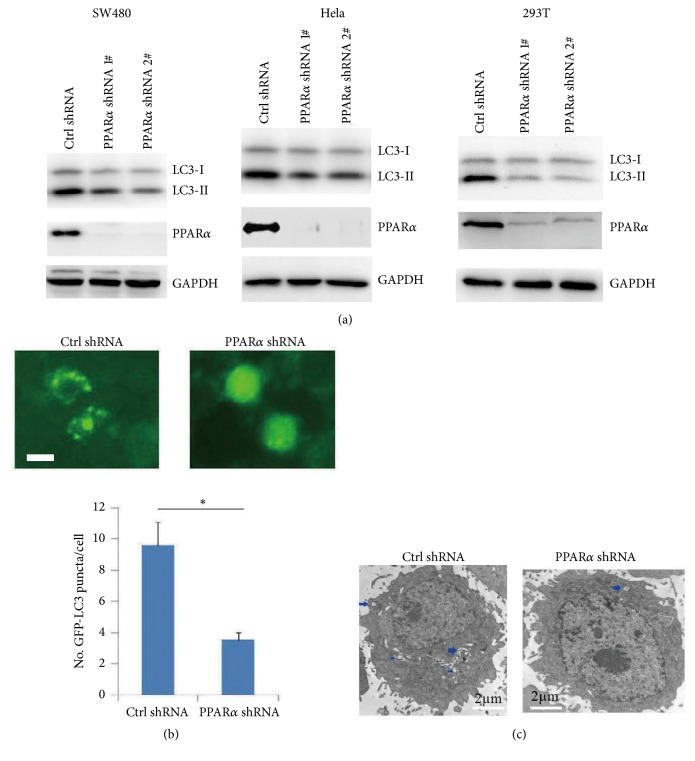
**Silenced PPAR** **α** **inhibits autophagy.** (a) PPAR*α* shRNA silenced SW480, Hela and 293T cell lysates were subjected to Western blot. (b) Representative images of GFP-LC3 puncta (autophagosomes) in PPAR*α* silenced SW480 cells. Scar bar: 20 *μ*m. The GFP-puncta was quantified. Results are expressed as means ± SEM (n=5). *∗P*<0.05. (c) TEM analysis of PPAR*α* shRNA silenced SW480 cells. Arrows show the autophagosomes.

**Figure 2:**
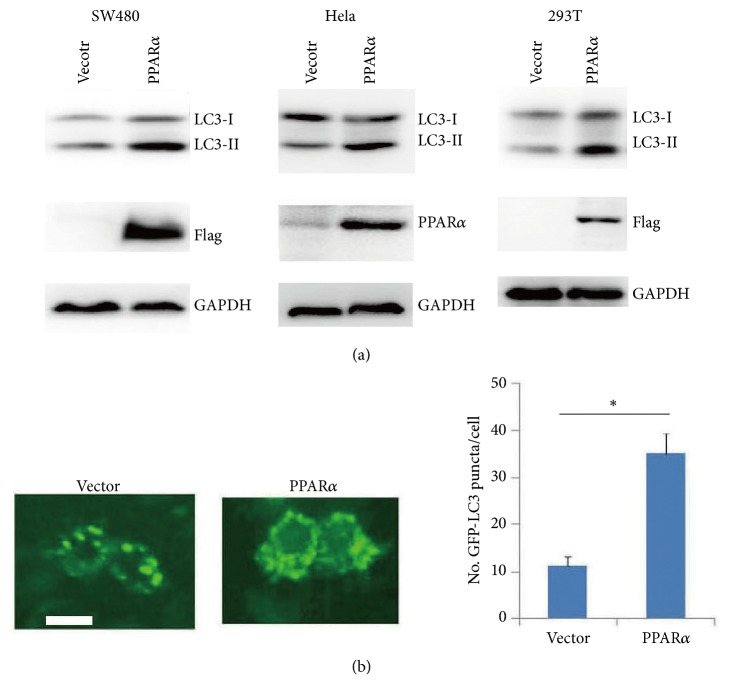
**Overexpression of PPAR** **α** **enhances autophagy**. (a) SW480 cells were transfected with vector or Flag-PPAR*α* plasmids for 36 h. Cell lysates were subjected to Western blot. (b) representative images of GFP-LC3 puncta (autophagosomes) in overexpression of PPAR*α* in SW480 cells. Scar bar: 20 *μ*m. The GFP-puncta was quantified. Results are expressed as means ± SEM (n=5). *∗P*<0.05.

Western blot analysis showed that PPAR*α* shRNA silence significantly decreased the LC3-II levels in SW480, Hela, and HEK293T cell lines (Figure 1(a)). Transfected GFP-LC3 plasmid in SW480 cells showed that PPAR*α* silence decreased autophagosome formation and GFP-LC3 puncta (Figure 1(b)), which was consistent with the transmission electron microscopy analysis (Figure 1(c)). Ligand-activated PPAR*α* promotes autophagy of AML12 cells or livers by inducing autophagy-associated gene expressions (LC3a, LC3b, etc.) [13]. To further detect whether PPAR*α*-mediated autophagy was involved in autophagy-associated gene expressions, qPCR analysis was performed. The results showed that PPAR*α* had no significant effect on autophagy-associated gene expressions (SFigure.  1). In contrast, overexpression of PPAR*α* in SW480 cells increased the LC3-II levels (Figure 2(a)) and GFP-LC3 puncta (Figure 2(b)), which had no effect on autophagy-associated gene expressions (SFigure.  2), suggesting that PPAR*α* promoted cancer cell autophagy independent of its transcription activity. Our previous results show that PPAR*α* induces the antiapoptotic Bcl2 protein ubiquitination and degradation [6]. Further analysis showed that PPAR*α* induced Bcl2 degradation, while it had no effect on the activation of caspase-3 in SW480 cells (SFigure.  3), suggesting that PPAR*α*-induced Bcl2 degradation had no effect on cancer cell apoptosis.

### 2.2. PPAR*α*-Mediated Bcl2 Degradation Increases Beclin-1/VPS34 Complex

**Figure 3:**
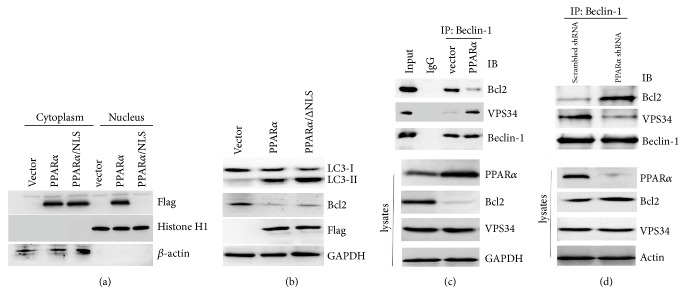
**PPAR** **α** **enhances Beclin-1/VPS34 complex formation.** (a) Extracts of cytoplasm and nucleus were subjected to Western blot by using overexpression of Flag-PPAR*α* or mutant plasmids in SW480 cells. (b) SW480 cells were transfected plasmids as indicated for 36 h. Cell lysates were subjected to Western blot. (c) SW480 cells were transfected with vector or Flag-PPAR*α* plasmids for 36 h. Cell lysates were subjected to immunoprecipitation and Western blot. (d) PPAR*α* shRNA silenced SW480 cell lysates were subjected to immunoprecipitation and Western blot.

Our results demonstrated that PPAR*α* induced cancer cell autophagy without effect on autophagy-associated gene expressions. To further detect PPAR*α* induced cancer cell autophagy independent of its transcription activity, the PPAR*α* nuclear location signal (NLS) was deleted and overexpressed in SW480 cells. The results showed that PPAR*α*/ΔNLS did not locate into nucleus by Western blot analysis (Figure 3(a)), while PPAR*α*/ΔNLS still induced autophagy (Figure 3(b)). These findings further demonstrated that PPAR*α*-mediated autophagy was independent of transcription activity, as Bcl2 interacts with Beclin-1 leading to disruption of Beclin-1/VPS34 complex and autophagy suppression [14]. Our previous finding shows that PPAR*α* acts as E3 ubiquitin ligase to induce Bcl2 ubiquitination and degradation [6]. Consistent with this, cytoplasmic PPAR*α* reduced Bcl2 protein levels corresponding to the increase in LC3-II levels (Figure 3(b)). To further detect whether Bcl2 degradation by PPAR*α* led to the increase in Beclin-1/VPS34 complex, immunoprecipitation analysis was performed. The results showed that overexpression of PPAR*α* increased the Beclin-1/VPS34 complex associated with reduction of Bcl2 protein levels (Figure 3(c)). In contrast, PPAR*α* shRNA silence reversed this event (Figure 3(d)), suggesting that PPAR*α*-mediated Bcl2 degradation increased the Beclin-1/VPS34 complex resulting in autophagy induction.

### 2.3. PPAR*α*/Autophagy Signaling Suppresses Tumor Progression

**Figure 4:**
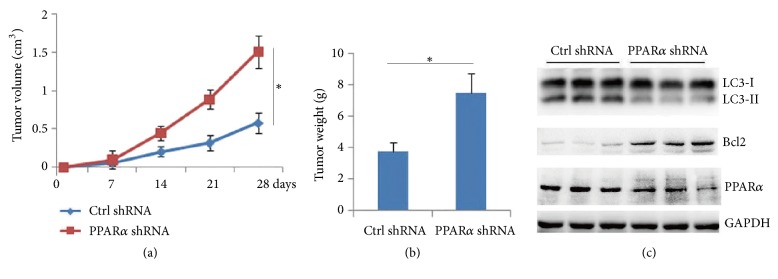
**PPAR** **α** **/autophagy signaling inhibits tumor progression.** Stabling expression of control or Flag-PPAR*α* shRNA SW480 cells were injected subcutaneously in nude mice for four weeks, and tumor volume (a) and tumor weight (b) were measured. Results are expressed as means ± SEM (n=5). **∗***P*<0.05. (c) Tumor lysates were subjected to Western blot.

To detect the effect of PPAR*α*-mediated autophagy on the tumor progression, xenograft tumor model was performed. The results showed that PPAR*α* shRNA silence promoted tumor growth (Figure 4(a)) and increased tumor weight (Figure 4(b)). Western blot analysis by using tumor lysates showed that silenced PPAR*α* reduced LC3-II levels and increased Bcl2 protein levels (Figure 4(c)). These findings showed that PPAR*α*-mediated autophagy suppressed tumor progression, which was involved in reduced Bcl2 protein levels.

### 2.4. PPAR*α* Agonist Enhances Autophagy-Mediated Tumor Suppression

**Figure 5:**
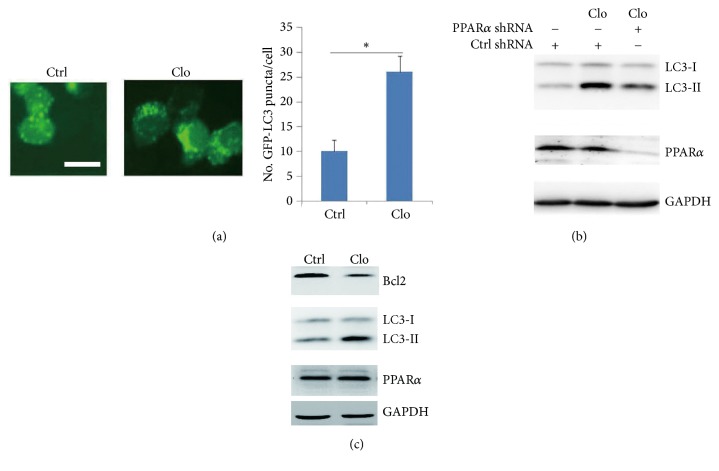
**Agonist enhances autophagy in a PPAR** **α** **-dependent manner.** (a) Representative images of GFP-LC3 puncta (autophagosomes) in 10*μ*M Clofibrate treated SW480 cells for 12 hours. Scar bar: 20 *μ*m. The GFP-puncta was quantified. Results are expressed as means ± SEM (n=5). **∗***P*<0.05. (b) SW480 cells were transfected with ctrl shRNA or PPAR*α* shRNA. After 36 h, cells were treated with 10*μ*M clofibrate for 12 hours. Cell lysates were subjected to Western blot. (c) SW480 cells were treated with 10*μ*M clofibrate for 12 hours. Cell lysates were subjected to Western blot. Data are triplicates from three independent experiments.

**Figure 6:**
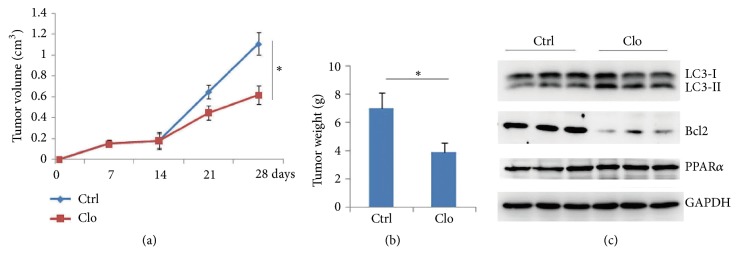
**Agonist of PPAR** **α** **inhibits tumor growth.** SW480 cells were injected subcutaneously in nude mice. After two weeks, mice were treated without or with Clofibrate (20mg/kg/day) for another two weeks by intraperitoneal injection. Tumor volume (a) and weight (b) were measured. Results are expressed as means ± SEM (n=5). **∗***P*<0.05. (c) Tumor lysates were subjected to Western blot.

SW480 cells were treated with PPAR*α* agonist (clofibrate); the results showed that clofibrate significantly increased autophagosome accumulation (Figure 5(a)). To further detect whether agonist-induced autophagy was PPAR*α* dependent, the PPAR*α* shRNA silenced SW480 cells were treated with clofibrate. The results showed that silenced PPAR*α* had no significant effect on LC3-II levels in response to clofibrate (Figure 5(b)), suggesting that clofibrate induced autophagy in a PPAR*α*-dependent manner. Further analysis showed that clofibrate reduced Bcl2 protein levels (Figure 5(c)). Moreover, agonist of PPAR*α* did not affect the autophagy-associated gene expressions (SFigure.  4). Xenograft mice model assay showed that PPAR*α* agonist clofibrate significantly inhibited tumor growth (Figure 6(a)) and tumor weight (Figure 6(b)). The* in vivo* tumor tissues further demonstrated that agonist clofibrate reduced Bcl2 protein levels and increased LC3-II levels (Figure 6(c)). These findings suggest that agonist enhanced autophagy-mediated tumor suppression in a PPAR*α*-dependent manner, which was not involved in autophagy-associated gene expressions.

### 2.5. PPAR*α*/Autophagy Signaling Increased Chemotherapy Sensitivity to Cancer Cells

**Figure 7:**
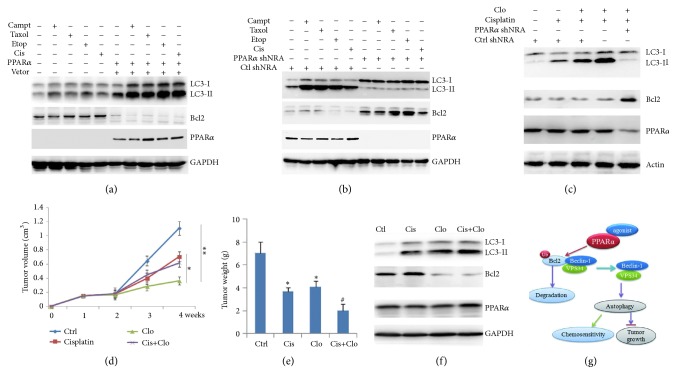
**PPAR** **α** **/autophagy enhances chemotherapy sensitivity to cancer cells.** (a) SW480 cells were transfected with vector or PPAR*α* plasmids for 36 h. After that, cells were treated with camptothecin (60*μ*M), taxol (300*μ*M), etoposide (600*μ*M), and cisplatinum (30*μ*M) for 6 h. Cell lysates were subjected to Western blot. (b) SW480 cells were transfected with ctrl shRNA or PPAR*α* shRNA plasmids for 36 h. After that, cells were treated with camptothecin (60*μ*M), taxol (300*μ*M), etoposide (600*μ*M), and cisplatinum (30*μ*M) for 6 h. Cell lysates were subjected to Western blot. (c) SW480 cells were transfected with ctrl shRNA or PPAR*α* shRNA plasmids for 36 h. After that, cells were treated 100*μ*M cisplatin or 100*μ*M cisplatin +10*μ*m clofibrate for 12 h. Cell lysates were subjected to Western blot. SW480 cells were injected subcutaneously in nude mice. After two weeks, mice were treated without or with cisplatin (3mg/kg), Clo (20mg/kg/day), or Clo (20mg/kg/day) +cisplatin (3mg/kg) for another two weeks by intraperitoneal injection. Tumor volume (d) or weight (e) was measured. Results are expressed as means ± SEM (n=5). **∗***P*<0.05, *∗∗P*<0.01. (f) Tumor lysates were subjected to Western blot. (g) The model of PPAR*α*/Bcl2/autophagy signaling inhibits tumor growth and chemotherapy sensitivity. Data are triplicates from three independent experiments.

To further detect the interaction of autophagy with chemotherapy drugs, SW480 cells were treated with chemotherapy drugs (camptothecin, taxol, etoposide, and cisplatin), the results showed that although these drugs increased LC3-II levels, overexpression of PPAR*α* significantly enhanced this event (Figure 7(a)). In contrast, PPAR*α* silenced cells inhibited chemotherapy drugs-induced autophagy (Figure 7(b)), suggesting that chemotherapy drugs induced autophagy in a PPAR*α*-dependent manner. The above data demonstrated that the ligand induced autophagy in a PPAR*α*-dependent manner. Consistent with this, clofibrate enhanced cisplatin-induced autophagy, which was terminated in PPAR*α* silenced SW480 cells (Figure 7(c)). These findings suggest that the agonist enhanced chemotherapy drugs-induced autophagy in a PPAR*α*-dependent manner.* In vivo* xenograft mice model assay showed that clofibrate together with cisplatin significantly inhibited tumor growth (Figure 7(d)) and reduced tumor weight (Figure 7(e)). The* in vivo* tumor tissues further demonstrated that agonist clofibrate/cisplatin significantly increased LC3-II levels (Figure 7(f)). These findings suggest that PPAR*α*/Bcl2/autophagy signaling increased chemotherapy sensitivity to cancer cells (Figure 7(g)).

## 3. Discussion

Autophagy is a conserved biochemical catabolic process that delivers cytoplasmic materials or organelles into lysosomes for degradation, which is also a progress of nutrient recycling [11]. Autophagy plays an important role in metabolic adaptation in cancer cell survival by digesting intracellular proteins and organelles in response to nutrient deprivation [11, 12]. Although autophagy increases cell survival under starvation stress, long-term autophagy without new nutrients replenishment leading to consumption of all available substrates and die (autophagy-associated cell death) [11, 12]. Therefore, autophagy is type II programmed cell death [12]. As a nuclear transcription factor, PPAR*α* plays an important role in regulating gene transcription. Other report shows that ligand-activated PPAR*α* increased autophagy of AML12 cells or livers via PPAR*α*-mediated autophagy-associated gene expressions such as LC3a and LC3b in liver cells or liver tissues [13]. However, the effect of PPAR*α* on cancer cell autophagy is still unclear. Here we found that PPAR*α* significantly induced cancer cell autophagy, while it was independent of its transcription activity. Similarly, the agonist enhanced cancer cell autophagy in a PPAR*α*-dependent manner, which was also independent of PPAR*α* transcription activity. These findings revealed a new mechanism of PPAR*α*-mediated cancer cell autophagy. As an antiapoptotic protein, Bcl2 interacts with Beclin-1 leading to disruption of Beclin-1/VPS34 complex and autophagy suppression [14]. As a proto-oncogene, Bcl2 inhibits cell apoptosis in the cancer development that is widely expressed in various malignancies such as lung, breast, prostate, and colorectal cancer [15], which plays a critical role in maintenance of normal tissues homeostasis and uncontrolled cell proliferation [12, 16]. Our previous findings show that PPAR*α* was independent of its transcriptional activity to induce Bcl2 ubiquitination and degradation [6]; here we found that PPAR*α* reduced cytoplasmic Bcl2 protein levels and increased LC3-II levels. Further analysis showed that PPAR*α*-mediated Bcl2 degradation led to increasing the Beclin-1/VPS34 complex formation, which promoted autophagy progression [14]. We further detected the relationship of PPAR*α*/Bcl2/autophagy signaling on tumor progression. Agonist (clofibrate) significantly decreased Bcl2 protein levels and increased autophagy and inhibition of tumor progression in a PPAR*α*-dependent manner, which suggests that PPAR*α* could be a potential drug target for cancer treatment. More importantly, chemotherapy drugs (camptothecin, taxol, etoposide, and cisplatin) were PPAR*α* dependent-induced autophagy formation; similar results observed that agonist/PPAR*α*/cisplatin signaling enhanced autophagy and subsequently promoted cancer cell chemotherapy sensitivity and tumor suppression. Taken together, PPAR*α*/Bcl2/autophagy signaling promoted autophagy and enhanced tumor suppression and chemotherapy sensitivity to cancer cells (Figure 7(g)).

## 4. Materials and Methods

### 4.1. Cell Culture, Reagents

The human embryonic kidney cell line HEK393T (ATCC® CRL-11268™), human colon cancer cell line SW480 (ATCC® CCL-228™), and human cervix cancer cell line Hela (ATCC® CCL-2™) were purchased from ATCC. These cells were cultured in DMEM supplemented with 10% fetal bovine serum (FBS, Gibco). Clofibrate was purchased from Toronto Research Chemical Inc. Taxol was purchansed from Ruibio. Cisplatin was purchased from Tokoyo Chemical industry. Etoposide and camptothecin were purchased from Hefei Bomei Biotechnology of China. Puromycin was purchased from Life Technologies.

### 4.2. Plasmids

Human PPAR*α* plasmid was described previously [7], which was mutated by the site-directed mutagenesis method. Plasmids were transfected by turboFect transfection reagent according to the manufacturer's instructions (Thermo Scientific). PPAR*α* shRNA plasmid was described previously [7].

### 4.3. Western Blot and Antibodies

LC3b antibody was purchased from Novus Biologicals. Actin, GAPDH, Bcl2, and PPAR*α* were purchased from Sangon Botech (Shanghai, China). Secondary antibodies were purchased from Jackson Immunoresearch. Western blot method was described previously [7, 17]. Data are triplicates from three independent experiments.

### 4.4. Quantitative Real Time PCR

Total RNA from SW480 cells was extracted by RNeasy kit (Sangon Biotech). The mRNA expressing levels were determined by Real-Time PCR analysis kit (Takara). The expression levels of relative mRNA were normalized against *β*-actin. Fold change over control was assayed by using the ΔCt method.

### 4.5. Transmission Electron Microscopy (TEM)

WT or PPAR*α* shRNA silenced SW480 cells were fixed in 2.5% glutaraldehyde for overnight at 4°C. Subsequently, samples were treated with 1% osmium tetroxide, embedded in resin. And then, the samples were cut into 70 nm sections for TEM analysis (Analytical and Testing Center of Nanjing Medical University, NJMU).

### 4.6. Xenograft Mice Model

The xenograft tumor model was described previously [17]. NU/NU nude mice (eight weeks, female) were obtained from SLAC Laboratory Animal Cooperation (Shanghai, China). The stable PPAR*α* silenced SW480 cells were selected by puromycin. SW480 cells (1x10^6^) were injected subcutaneously in the nude mice. Tumor volume was measured every week by using a digital caliper during four weeks. In addition, SW480 cells (1x10^6^) were injected subcutaneously in nude mice. After two weeks, mice were treated without or with Clo (20mg/kg/day) or Clo+cisplatin (3mg/kg) for another two weeks by intraperitoneal injection. Tumor volume = 1/2(length × width^2^). All studies were carried out with the approval of the Jiangsu University Animal Care Committee.

### 4.7. Statistical Analysis

Data are expressed as the mean ± SEM. Statistical comparison was carried out with student's t test or one way analysis of variance (ANOVA) and Dunnett's test as appropriate.

## Data Availability

The data used to support the findings of this study are available from the corresponding author upon request.

## Supplementary Materials

Supplementary Materials SFigure.1. Silenced PPAR*α* had no effect on the autophagy-associated gene expressions. SW480 cells were transfected control shRNA or PPAR*α* shRNA for 36 h. mRNA was extracted for qPCR analysis. Results are expressed as means ± SEM (n=4). SFigure.2. Overexpression of PPAR*α* had no effect on the autophagy-associated gene expressions. SW480 cells were transfected with vector or PPAR*α* plasmid for 36 h. mRNA was extracted for qPCR analysis. Results are expressed as means ± SEM (n=4). SFigure. 3. PPAR*α*-induced Bcl2 degradation had no effect on apoptosis. SW480 cells were transfected with control vector or Flag-PPAR*α* for 36 h. One group was treated with MG132 (20*μ*M) for 6 h before cell lysis. The other group was treated with cisplatinum (30*μ*M) for 6 h before cell lysis. Cell lysates were subjected to Western blot. SFigure.4. Clo had no effect on the autophagy-associated gene expressions. SW480 cells were treated with or without Clo for 12 h. mRNA was extracted for qPCR analysis. Results are expressed as means ± SEM (n=4).

## References

[1] Michalik L., Desvergne B., Wahli W. (2004). Peroxisome-proliferator-activated receptors and cancers: complex stories. *Nature Reviews Cancer*.

[2] Hou Y., Moreau F., Chadee K. (2012). PPAR*γ* is an E3 ligase that induces the degradation of NF*κ*B/p65. *Nature Communications*.

[3] Hou Y., Gao J., Xu H. (2014). PPAR*γ* E3 ubiquitin ligase regulates MUC1-C oncoprotein stability. *Oncogene*.

[4] Zhang Z., Xu Y., Xu Q., Hou Y. (2013). PPAR*γ* against tumors by different signaling pathways. *Onkologie*.

[5] Gao J., Yuan S., Jin J., Shi J., Hou Y. (2015). PPAR*α* regulates tumor progression, foe or friend?. *European Journal of Pharmacology*.

[6] Gao J., Liu Q., Xu Y. (2015). PPAR*α* induces cell apoptosis by destructing Bcl2. *Oncotarget *.

[7] You M., Jin J., Liu Q., Xu Q., Shi J., Hou Y. (2017). PPAR*α* promotes cancer cell Glut1 transcription repression. *Journal of Cellular Biochemistry*.

[8] Baker B. G., Ball G. R., Rakha E. A. (2013). Lack of expression of the proteins GMPR2 and PPAR*α* are associated with the basal phenotype and patient outcome in breast cancer. *Breast Cancer Research and Treatment*.

[9] Pancione M., Forte N., Sabatino L. (2009). Reduced *β*-catenin and peroxisome proliferator–activated receptor–*γ* expression levels are associated with colorectal cancer metastatic progression: correlation with tumor-associated macrophages, cyclooxygenase 2, and patient outcome. *Human Pathology*.

[10] Pattingre S., Tassa A., Qu X. (2005). Bcl-2 antiapoptotic proteins inhibit Beclin 1-dependent autophagy. *Cell*.

[11] Kaur J., Debnath J. (2015). Autophagy at the crossroads of catabolism and anabolism. *Nature Reviews Molecular Cell Biology*.

[12] Hotchkiss R. S., Strasser A., McDunn J. E., Swanson P. E. (2009). Cell death. *The New England Journal of Medicine*.

[13] Lee J. M., Wagner M., Xiao R. (2014). Nutrient-sensing nuclear receptors coordinate autophagy. *Nature*.

[14] Decuypere J. P., Parys J. B., Bultynck G. (2012). Regulation of the autophagic bcl-2/beclin 1 interaction. *Cells*.

[15] Kaklamanis L., Savage A., Mortensen N. (1996). Early expression of bcl‐2 protein in the adenoma–carcinoma sequence of colorectal neoplasia. *The Journal of Pathology*.

[16] Hou Y., Gao F., Wang Q. (2007). Bcl2 impedes DNA mismatch repair by directly regulating the hMSH2-hMSH6 heterodimeric complex. *The Journal of Biological Chemistry*.

[17] Zhang W., Xu Y., Xu Q., Shi H., Shi J., Hou Y. (2017). PPAR*δ* promotes tumor progression via activation of Glut1 and SLC1-A5 transcription. *Carcinogenesis*.

