# Supplementary material for: PPARα Enhances Cancer Cell Chemotherapy Sensitivity by Autophagy Induction
Source: J Oncol. 2018 Nov 4;2018:6458537. doi: 10.1155/2018/6458537 (PMC6241347; doi:10.1155/2018/6458537)
Supplement: Supplementary Materials — SFigure.1. Silenced PPARα had no effect on the autophagy-associated gene expressions. SW480 cells were transfected control shRNA or PPARα shRNA for 36 h. mRNA was extracted for qPCR analysis. Results are expressed as means ± SEM (n=4). SFigure.2. Overexpression of PPARα had no effect on the autophagy-associated gene expressions. SW480 cells were transfected with vector or PPARα plasmid for 36 h. mRNA was extracted for qPCR analysis. Results are expressed as means ± SEM (n=4). SFigure. 3. PPARα-induced Bcl2 degradation had no effect on apoptosis. SW480 cells were transfected with control vector or Flag-PPARα for 36 h. One group was treated with MG132 (20μM) for 6 h before cell lysis. The other group was treated with cisplatinum (30μM) for 6 h before cell lysis. Cell lysates were subjected to Western blot. SFigure.4. Clo had no effect on the autophagy-associated gene expressions. SW480 cells were treated with or without Clo for 12 h. mRNA was extracted for qPCR analysis. Results are expressed as means ± SEM (n=4). [file 6458537.f1.docx]

**Supplementary legends and Figure**


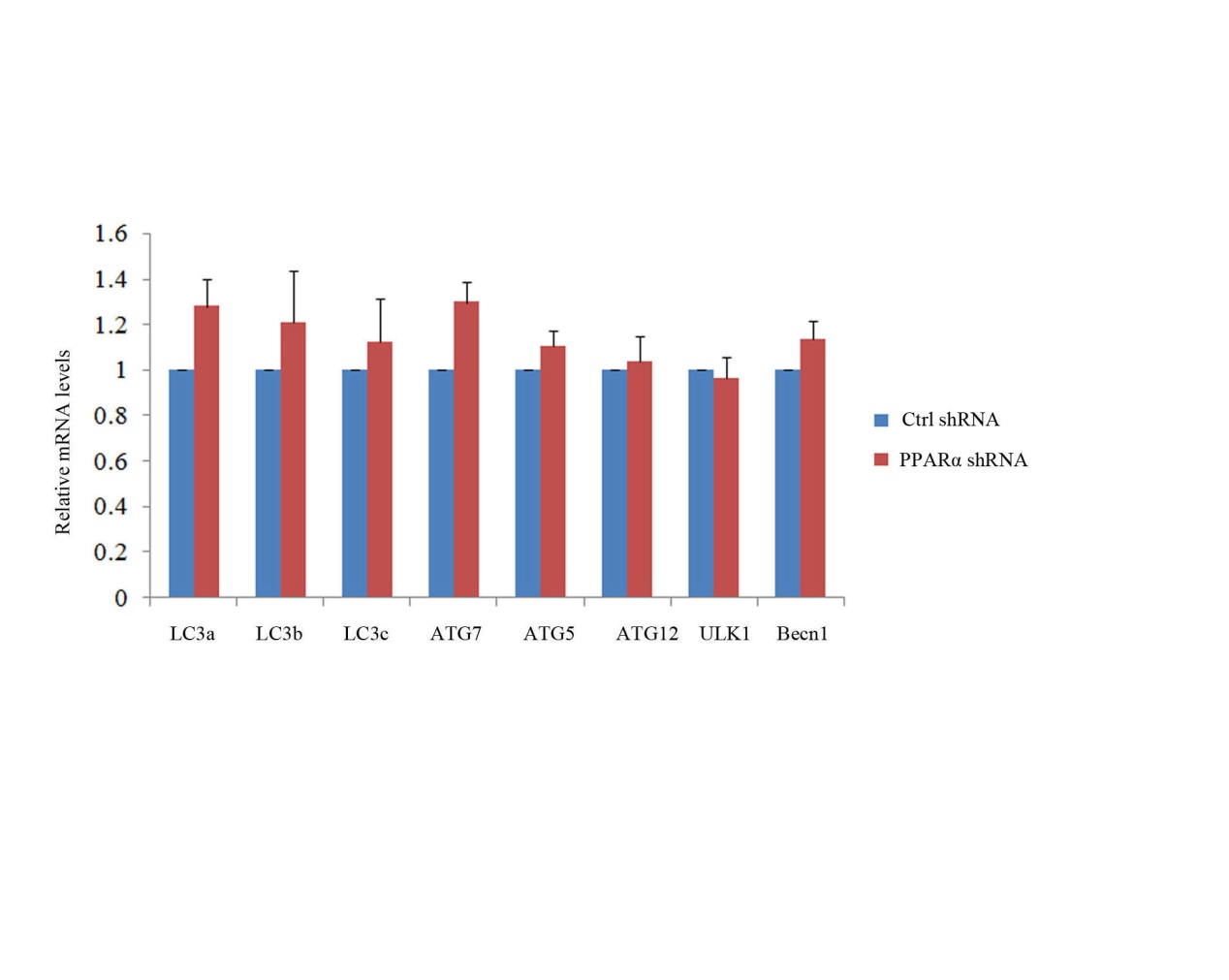


**SFigure.1.**  **Silenced PPARα had no effect on the autophagy-associated gene expressions.**

SW480 cells were transfected control shRNA or PPARα shRNA for 36h. mRNA was extracted for qPCR analysis. Results are expressed as means ± SEM (n=4).

**
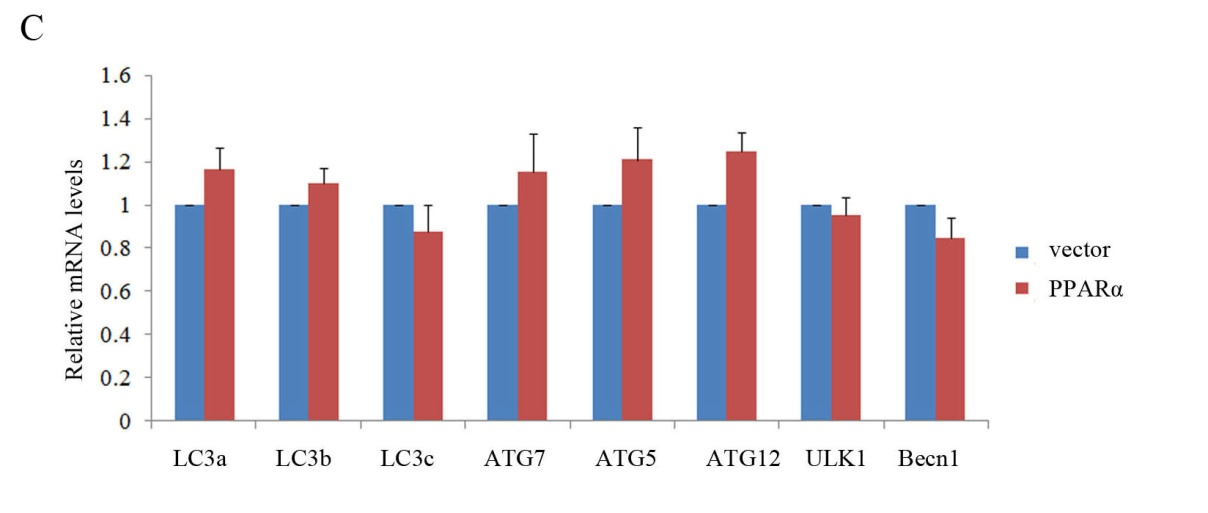
**

**SFigure.2.**  **Overxpression of PPARα had no effect on the autophagy-associated gene expressions.**

SW480 cells were transfected with vector or PPARα plasmid for 36h. mRNA was extracted for qPCR analysis. Results are expressed as means ± SEM (n=4).

**
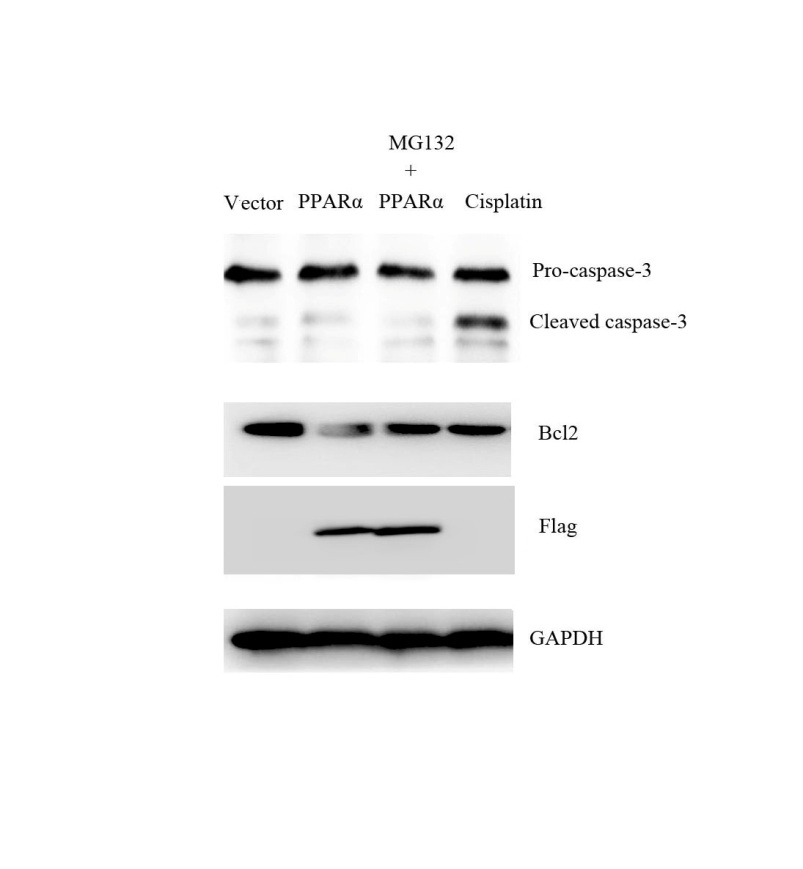
**

**SFigure. 3. PPARα-induced Bcl2 degradation had no effect on apoptosis.**

SW480 cells were transfected with control vector or Flag-PPARα for 36h. One group was treated with MG132 (20μM) for 6h before cell lysis. The other group was treated with cisplatinum (30µM) for 6h before cell lysis. Cell lysates were subjected to Western blot.


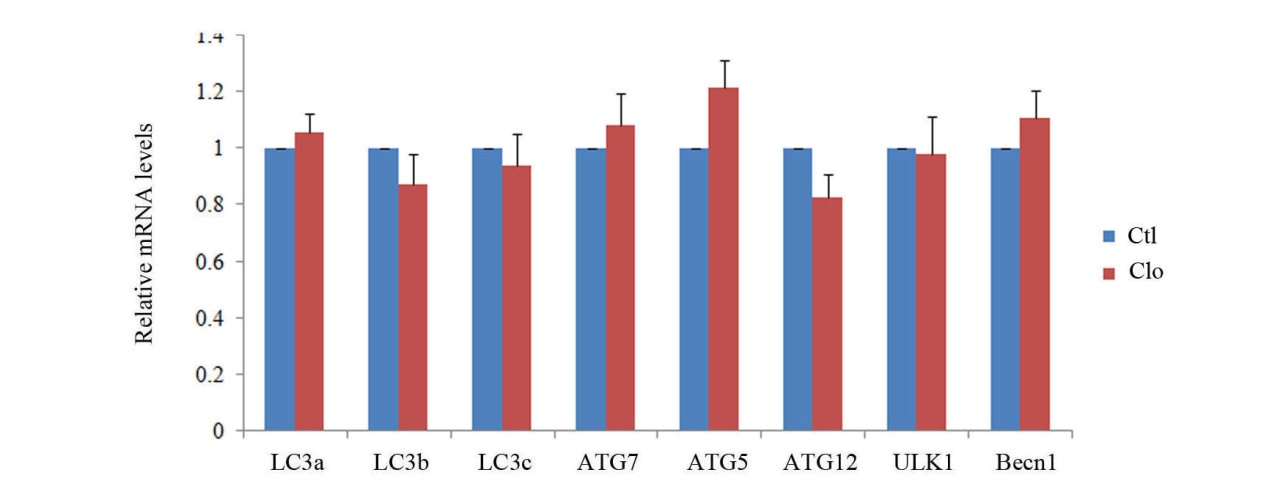


**SFigure.4.**  **Clo had no effect on the autophagy-associated gene expressions.**

SW480 cells were treated with or without Clo for 12h. mRNA was extracted for qPCR analysis. Results are expressed as means ± SEM (n=4).
